# Occupational Risk Factors for SARS-CoV-2 Infection in Hospital Health Care Workers: A Prospective Nested Case-Control Study

**DOI:** 10.3390/life12020263

**Published:** 2022-02-09

**Authors:** Alex Dusefante, Corrado Negro, Pierlanfranco D’Agaro, Ludovica Segat, Antonio Purpuri, Luca Cegolon, Francesca Larese Filon

**Affiliations:** 1 Clinical Unit of Occupational Medicine, Department of Medical, Surgical & Health Sciences, University of Trieste, 34129 Trieste, Italy; corrado.negro@asugi.sanita.fvg.it (C.N.); antoniopurpuri1987@gmail.com (A.P.); larese@units.it (F.L.F.); 2 Hygiene & Public Health Clinical Unit, Department of Medical, Surgical & Health Sciences, University of Trieste, 34129 Trieste, Italy; pdagaro@units.it; 3 Hygiene & Public Health Clinical Unit, Department of Laboratory, University Health Agency Giuliano-Isontina (ASUGI), 34129 Trieste, Italy; ludovica.segat@units.it; 4 Public Health Department, University Health Agency Giuliano-Isontina (ASUGI), 34129 Trieste, Italy

**Keywords:** SARS-CoV-2, COVID-19, occupational, risk factors

## Abstract

**Introduction**: Health Care Workers (HCWs) are at a particular high risk of SARS-CoV-2 infection due to direct and indirect exposure to COVID-19 patients and Aerosol-Generating Procedures (AGPs). The aim of the study was to assess the risk factors for SARS-CoV-2 infection in HCWs exposed to COVID-19 patients, to evaluate the adherence and effectiveness of Infection Prevention and Control (IPC) measures, to describe the clinical presentation for SARS-CoV-2 infection in HCWs and to determine serological responses in HCWs. **Methods**: HCWs exposed to COVID-19 patients during the previous 14 days with a confirmed case status were recruited as cases; HCWs exposed to COVID-19 patients during the previous 14 days in the same ward without a suspected/probable/confirmed case status were recruited as controls. Serum samples were collected as soon as possible and after 21–28 days from all participants. Data were collected with a WHO standardized questionnaire as soon as possible and after 21–28 days. **Results**: All social, occupational and personal variables considered were not associated with an increased risk of SARS-CoV-2 infection. **Conclusions**: Our study showed a high knowledge of IPC measures and very high PPE use among HCWs.

## 1. Introduction

Severe Acute Respiratory Syndrome Coronavirus 2 (SARS-CoV-2), a novel strain of Coronavirus, is responsible of Coronavirus Disease 19 (COVID-19), a potentially severe and fatal respiratory illness. First discovered in 2019, SARS-CoV-2 has become one of the most important health emergencies of the 21st century [1]. As of 5 January 2022, SARS-CoV-2 has infected 295,309,551 individuals worldwide with a total of 5,472,624 attributable deaths [2,3], although some evidence suggests that the actual number of infections could be up to 18 times higher [4]. Italy was severely hit by the first wave of the SARS-CoV-2 pandemic and, as of 5 January 2022, 6,566,947 people have been infected, with 138,045 total attributable deaths [2,3] and 150,492 infections (2.96%) affecting Health Care Workers (HCWs) [5].

HCWs represent the frontline response to the SARS-CoV-2 pandemic and thus have a high risk of acquiring the infection, due to exposure to COVID-19 patients and Aerosol-Generating Procedures (AGPs) [6,7,8,9,10]. HCWs tend to have a high incidence of infection, with less severe illness and mortality compared to the general population, mainly due to lower mean age and low comorbidities prevalence [10]. Nurses, HCWs working in hospitalization/emergency wards and infectious diseases personnel seem to have the highest risk of infection, with ob-gyn personnel and pediatrics personnel having the lowest risk [10,11,12]. By contrast, the evidence regarding anesthesiologist/intensivists is inconclusive [11,12,13,14,15].

Occupational risk factors for SARS-CoV-2 infection in HCWs have been extensively studied [1,6,7,8,9,10,11,12,13,14,15,16,17,18,19,20,21,22,23,24,25,26,27,28,29,30,31,32,33], mainly focusing on job task, ward type, personal protective equipment (PPE) usage, AGPs exposure, comorbidities and exposure in their social life outside their work environment. Data on Infection Prevention and Control (IPC) measures formation/adherence and studies focusing on ascertained occupational SARS-CoV-2 infections among COVID-19-dedicated HCWs are scarce.

## 2. Methods

The Clinical Unit of Occupational Medicine at the University of Trieste (north-eastern Italy) participated to the WHO project “*Assessment of risk factors for coronavirus disease 2019 (COVID-19) in health workers: protocol for a case-control study*” with 2 sites, “Maggiore Hospital” and “Cattinara Hospital”. The former hospital only provides outpatient services, whereas the latter has hospital wards and an operating theatre, mainly providing inpatient services.

The WHO protocol and key adjustments made for our local data collection and analysis will be presented. The full study design is accessible elsewhere [34].

The main objective of this nested case-control study was to assess the risk factors for SARS-CoV-2 infection in HCWs exposed to COVID-19 patients. Secondary outcomes were:

– to evaluate the adherence and effectiveness of current COVID-19 IPC measures;
– to describe the clinical presentation of SARS-CoV-2 infection in HCWs;
– to determine the serological responses in HCWs with confirmed SARS-CoV-2 infection following exposure to COVID-19 patients, and in HCWs exposed to COVID-19 patients but without SARS-CoV-2 infection.

HCWs were defined as any member of staff in the health care facility directly or indirectly involved in the care of COVID-19 patients. Exposure to COVID-19 patients was defined as close contact (within 1 m and for more than 15 min) with a suspected/probable/confirmed COVID-19 patient or indirect contact with fomites or with materials, devices or equipment linked to a suspected/probable/confirmed COVID-19 patient(s).

A case was defined as a HCW exposed to a COVID-19 patient in a health care setting during the 14 days before testing positive for COVID-19. The exclusion criterion was having a confirmed COVID-19 case among close contacts (friends or household members), in the previous 14 days.

A control was defined as a HCW exposed to a COVID-19 patient in a health care setting during the 14 days before entering this study and not being classified as a suspected, probable or confirmed COVID-19 case. Two to four controls were recruited per each case, according to WHO protocol [34].

A total of 120 study participants were recruited, broken down by 29 (24.2%) cases vs. 91 (75.8%) controls.

Any HCW who was defined as a confirmed COVID-19 case was approached by telephone by a WHO-trained occupational medicine trainee and screened for potential exclusion criteria. Controls were selected by convenience sampling from a list of all HCWs in the same ward with any exposure to COVID-19 patients, approached over the phone and screened for inclusion and exclusion criteria. Verbal and written informed consent was obtained both from cases and controls.

Demographic information, symptom severity, medical history, use of medications, availability of PPE, adherence to IPC measures and contact with and exposure to COVID-19 patients was collected from all participants following their admission to the health care facility. A first serum sample was collected as soon as possible. A follow-up completion form was used to investigate HCWs’ health status and symptom severity after 21–28 days. A second serum sample was collected as soon as possible.

There was missing information on blood samples for two cases at T1 and 7 controls at T2.

Viral RNA was extracted form nasopharyngeal specimens and determined by RT-PCR targeting the E, N and RdRp gene of SARS-CoV-2 according to the Center for Disease Control and Prevention (CDC) and Charitè laboratory protocols [35]. The cycle threshold (CT) values of RT-PCR were used as qualitative indicators of SARS-CoV-2 RNA viral load in specimens, with lower CT values corresponding to higher viral copy numbers. A CT value < 30 was considered positive for SARS-CoV-2 RNA.

Peripheral venous blood samples were obtained and stored at +4 °C until processing. Sera were tested for SARS-CoV-2 antibodies using the LIAISON SARS-CoV-2 Trimerics IgG assay (Liason, DiaSorin, Stillwater, MN, USA), a quantitative assay for the detection of IgG antibodies anti-Trimeric Spike glycoprotein of SARS-CoV-2, with a positive cut-off ≥33.8 BAU/mL and an assay range of 4.81–2080 BAU/mL. Clinical sensitivity was reporedly 98.7% (95% IC 94.5—99.6%) 15 days after PCR and specificity was 99.5% (95% CI: 99.0–99.7%). Sera were additionally tested with ELISA assay Wantai SARS-CoV-2 Ab ELISA (Beijing Wantai Biological Pharmacy Enterprise). Samples with a cutoff ratio (absorbance of the sample at 450 nm divided by 0.19) ≥1 AU/mL were considered positive [36].

### Statistical Analysis

Statistical analyses were performed using STATA v16.0 (Stata Corp LCC, Lakeway Drive, TX, USA). Continuous variables were expressed by median and interquartile range (IQR), while categorical terms by absolute and relative frequency. Differences between groups were assessed by Mann–Whitney test for continuous variables and Pearson’s chi-square test for categorical variables.

Risk factors for those with SARS-CoV-2 infection were investigated with univariable and multivariable logistic regression analysis. A *p*-value < 0.05 was set for statistical significance.

The study was approved by the local Ethical Committee of Friuli-Venezia Giulia Region (CEUR, ethical approval code n 083_2020, 16 September 2020).

## 3. Results

From 1st September 2020 to 31 January 2021, 12,080 COVID-19 cases, 1532 hospital admissions due to COVID-19 and 395 COVID-19 related deaths were recorded in Trieste (data not shown). During this time period, 120 participants (29 cases and 91 controls) were recruited. General, occupational and social characteristics of the study populations are presented in Table 1, by cases with controls. There was no statistically significant difference between cases and controls for all considered variables.

The study population comprised 70 females (58.3%) and 50 males (41.7%). The median age was 40 years (IQR: 32; 50). In total, 85 HCWs (70.8%) had a university degree or higher level of education and 35 (29.2%) had junior/senior secondary school education.

Half of the study population were registered nurses (50.0% = 60/120), 20.8% (=25/120) assistant nurses, 12.5% (=15/120) patient transporters, 10.8% (=13/120) medical doctors and a few other medical professionals (radiology technicians, physiotherapists, admission clerks).

Out of the total, 49 (40.8%) HCWs were assigned to COVID-19 patients’ care, with a median of 10 (IQR 10–12) days dedicated to COVID-19-ward during the previous 2 weeks. Eighty-six HCWs (71.7%) reported having received a COVID-19 specific training; further details regarding duration and training method are presented in Table 1. The majority of our study population reported no use of public transport and no social interactions during the previous 14 days (86–71.4% and 73–60.8% respectively); further details can be seen in Table 1.

Table 2 presents the knowledge of and adherence to IPC measures among the study participants. We found no statistically significant difference between cases and controls. Knowledge and adherence were close or equal to 100% for the majority of investigated items (moments of hand hygiene, PPE usage and availability, alcohol-based hand rub availability, IPC standard precautions).

Characteristics of HCWs’ exposure to COVID-19 patients are described in Table 3.

No statistically significant difference between cases and controls was found regarding exposure to COVID-19 patients, number and duration of close contacts, number of face-to-face exposures, PPE usage during face-to-face exposure, adherence to respirators fit-test protocols, gloves removal protocols and exposure to COVID-19 patients’ body fluids, materials and surroundings. We found a statistically significant difference between cases and controls regarding exposure to AGPs: 51.6% of controls vs 27.6% of cases had exposure to AGPs during the 14 days before study entry (*p* = 0.021).

Table 4 presents the clinical symptoms reported by male and female cases at T1.

Only 1 case (3.4%) remained asymptomatic. The most common symptoms were fever and cough (14 cases, 48.3%), followed by myalgia (13 cases, 44.8%), anosmia/dysgeusia (12 cases, 41.4%), runny nose and fatigue (7 cases, 24.1%), sore throat and headache (6 cases, 20.7%); other symptoms had a lower prevalence (Table 4). No statistical test was used to assess differences by sex due to low statistical power.

Data regarding comorbidities and medication intake are presented in Table 5. No statistically significant difference was found between cases and controls. The majority of study subjects reported no comorbidities (73.3% = 88/120) and no regular medication intake (70.0% = 84/120).

One HCW (3.5% of cases) was hospitalized for a SARS-CoV-2-related pneumonia; no HCW died by the end of the study.

Median and IQR serology values of chemoluminescence immunoassay (CLIA, expressed in BAU/mL) and enzyme-linked immunosorbent assay (ELISA, expressed in optical density value) between cases and controls at T1 and T2 are shown in Table 6. Whilst CLIA levels were quantified only on positive test results, ELISA levels measured on all samples. A statistically significant difference between cases and controls was found both for CLIA (*p* = 0.02) and ELISA assay (*p* < 0.001) between T1 and T2.

Personal and occupational factors associated with SARS-CoV-2 infection were investigated by univariable and multivariable logistic regression analysis. As can be seen from Table 7, no significant association with SARS-CoV-2 infection was found for all variables included in the analysis. However, at univariable analysis, a longer duration (≥2 h) of COVID-19 training was associated with significantly lower risk of SARS-CoV-2 infection (OR = 0.41; 95% CI: 0.17; 0.89).

## 4. Discussion

### 4.1. Main Findings

The present study evaluated the personal, social and occupational risk factors for SARS-CoV-2 infection in a cohort of 120 HCWs exposed to COVID-19 patients. There was no difference between cases and controls regarding sex, age, occupation, educational level, number of days dedicated to COVID-19-wards, IPC training (method and duration), social interactions and public transport use. The risk of SARS-CoV-2 infection was not associated with sex of respondents. 

### 4.2. Interpretation of Findings in Relation to Other Studies

Whilst a nasopharyngeal swab (NPS) positivity [1,8] and seropositivity [4,12,18] similar to the present study was already reported elsewhere, some studies [15,16,36] found a higher risk of seropositivity for males or females [19]. In contrast with some studies [8,30,32,37] we found no association between age and risk of SARS-CoV-2 infection. This result may be explained by the low median age and small IQR of the study population (40 years; IQR 32–52; with no difference between cases and controls).

Furthermore, occupation was not a significant risk factor in the present study, which is in line with some studies investigating the risk of both NPSs positivity [32] and seropositivity [18,23,38,39]. Some authors identified a decreased risk for doctors and nurses and an increased risk for technicians and sanitation workers [16,28,40] although another study from Trieste teaching hospitals found a higher risk of SARS-CoV-2 infection in nurse aides/auxiliary personnel and a lower risk for technicians [41]. The lack of significance could be related to our inclusion criteria, which only allowed recruitment of HCWs exposed to COVID-19 patients, thus exposed to high risk of infection regardless of job task. The job task at highest risk was registered nurse (48.3%), as already reported in a systematic review on 97 studies comprising 230,398 HCWs [10]. No risk of SARS-CoV-2 infection was found for COVID-19 wards’ dedicated personnel compared with HCWs providing care to COVID-19 patients in non-COVID-19 wards or with HCWs temporarily working in COVID-19 wards. We believe this might be due to exposure to COVID-19 patients, their fomites and surroundings being similar between these groups [31].

In contrast with the current literature’s evidence [8,20,27], no significant decreased risk of infection was found in HCWs who received a specific training for COVID-19 patients’ care, regardless of its type and duration. Nevertheless, at univariable analysis HSWs receiving longer (≥2 h) COVID-9 training were less likely to be infected SARS-CoV-2. It is possible that a larger study sample might confirm the latter result also at multivariable analysis.

In total, 27.6% of our study population reported not having received any training on PPE use and care for COVID-19 patients; in a UK study 22.5% (=1382/6152) HCWs aged ≥18 from primary and secondary care services reported lack of access to PPE while managing confirmed COVID-19 cases during the first pandemic wave (1 February to 25 May 2020) [8]. However, the latter figures may have likely changed since the early days of the pandemic. Although some lack of information could be related to logistical difficulties in implementing an organized training before risk exposure during an emergency situation, the majority of these missing data may be attributable to recall bias, considering the optimal results of the IPC measures questionnaire (as described below). This bias could be responsible for the absence of statistical significance.

No significant correlation was found between public transport usage or social interactions in the previous 14 days and risk of SARS-CoV-2 infection. The majority of the study population did not use public transport (71.4%) and did not have any social interaction outside the work environment (60.5%). It is well known that SARS-CoV-2 transmission is high in crowded places [38]. Very high PPE usage and IPC measures adherence among the study population (as described below), even outside the worksite, may explain the lack of infection risk.

Knowledge of and adherence to IPC measures were close to 100% for all investigated items; similar results were obtained using the same questionnaire and a similar study design in a case-control study on 256 HCWs in a tertiary care hospital in India [29]. These results were obtained through a standardized questionnaire and not by direct observation; a recall bias should therefore be taken into account. Nevertheless, it is well known that HCWs are highly compliant with PPE use compared with white collar workers or the general population [38]. Thus, these results represent an indirect indication of good information and training of HCWs on standard IPC measures.

The main determinants of HCWs’ exposure to COVID-19 patients were investigated in the present study. Exposure to AGP was associated with a decreased risk of SARS-CoV-2 positivity at NPS (*p* = 0.02), although this result was not confirmed ay univariable and multivariable logistic regression analysis in the present study. Nevertheless, the latter finding has already been thoroughly investigated in the open literature [6,7,8,9,13,14,21,42]. AGPs are commonly associated with a high risk of respiratory viruses’ infection [21]: a systematic review and meta-analysis on 17 studies reported an OR of SARS-CoV-2 infection of 6.69 for endotracheal intubation, 3.65 for Non-Invasive Ventilation and 10.03 for nebulized medications [9]. Nonetheless, there is inconclusive evidence regarding viability and infectivity of SARS-CoV-2 in aerosols and uncertainty as to the nature and impact of aerosol transmission and its contribution compared with other modes of transmission [42], since coughing, increased work of breathing, increased closing capacity and altered respiratory lining fluid generate more pathogenic aerosol than AGPs [7]. HCWs regularly exposed to AGPs have been shown to be at a lower risk of infection (OR = 0.50; 95% CI 0.21–1.2) [8,15] probably because of high level of precautions observed, strict use of PPE and lower infectiousness of patients [13].

The number of COVID-19 patients assisted during the previous 14 days, the number of close contacts <1 m during the previous 14 days, the maximum duration of close contacts and face-to-face exposures were not significantly different between cases and controls. The exposures to COVID-19 patients’ body fluids, materials and surroundings were also not associated with an increased risk of infection. The current literature underlines the increased risk of SARS-CoV-2 infection for HCWs caring for COVID-19 patients for the majority of days (OR = 1.32; 95% CI 0.56–3.1) or in situation with increased exposure to the virus (OR = 1.1; 95% CI 0.34–3.3) [20,22,31]. The latter findings may be explained by the very high level of compliance with PPE usage and IPC measures in the present study population, already reported elsewhere [9,33]. Indeed, 100% of HCWs reported having always used adequate PPE during face-to-face exposure, 85% reported always test-fitting the respirator upon donning and 93.8% reported removing gloves after every contact with COVID-19 patients (while the remaining reported an adequate hygienization of gloves with alcohol-based hand rub and adequate doffing upon exiting the patients’ room).

The vast majority (96.6% = 28/29) of our cases were symptomatic. Anosmia, fever, myalgia and cough were the most frequently reported symptoms among our cases, with slightly lower prevalence than those reported in a systematic review and meta-analysis on 97 studies and 230,398 HCWs. Similar results were also reported in an observational retrospective cohort study on 4,632 HCWs from a COVID-19 referral center in Northern Italy [1]. Anosmia, fever and myalgia are in fact the main symptoms associated with SARS-CoV-2 positivity in HCWs. One HCW (3.5% of cases) was hospitalized due to SARS-CoV-2 related pneumonia in the present study, in line with current figures of severe COVID-19 among HCWs (5%) [10]. No HCW died by the end of the present study.

No association was found between underlying medical conditions or medication use and SARS-CoV-2 infection. As can be seen from Table 4, prevalence of comorbidities (26.9%, with no difference between cases and controls) was indeed lower than what is reported in the open literature [10] and might be attributable to selection bias: Italian law requires regular check-ups by occupational doctors to evaluate fitness for work and thus no HCW with risk factors for increased susceptibility to biological agents can be exposed to COVID-19 patients. Prevalence of medication intake was also low in the present study (30.3%, with no difference between cases and controls, see Table 5 for more details), probably for similar reasons: no HCWs using medications that can reduce immunological responses can be exposed to COVID-19 patients. Although we did not find any correlation between comorbidities or medication intake and SARS-CoV-2 infection, the pooled analysis of all WHO sites may be sufficiently powerful to detect potential risk factors.

Finally, CLIA serology and ELISA optical density were significantly different between cases at T1 and T2 and controls, signifying a reliable diagnosis of SARS-CoV-2 infection and accurate selection of controls.

### 4.3. Strengths and Limitations

As already discussed, a limitation of the present study is that most of the data were collected through a WHO standardized questionnaire, not by direct observation or by using objective measures (e.g., hospitalization records, PPE supply and disposal registries, HCWs personal medical records), therefore recall bias shall not be ruled out. This limitation might be offset by the fact that all questionnaires were administered by the same occupational medicine trainee. Moreover, the use of a standardized questionnaire enables WHO to pool standardized data from all sites regardless of technical, economical and logistical differences.

Another study limitation lies in the limited availbility of susceptibe controls, naive from past SARS-CoV-2 infection, since both teaching hospitals in Trieste were severely hit by the first two pandemic waves, with several internal outbreaks and a large number of HCWs infected. These HCWs, once recovered, were still working in the COVID-19 patients’ care at the time of our study but could not be recruited as controls because of exclusion criteria, thus sometimes severely limiting the number of potential controls working in the same ward as the corresponding case.

Despite the above limitations, the present study has also relevant strengths: all cases and controls were recruited, interviewed and followed up by the same WHO-trained occupational medicine doctor, thus limiting interpersonal variability of the interviewer. Secondly, since HCWs with extraprofessional contact with suspected/confirmed SARS-CoV-2-infected individuals were excluded, non-occupational or household infections were ruled out. Moreover, the use of a standardized questionnaire with the limitations previously described enabled an indepth evaluation of a very large number of personal, social and occupational factors influencing HCWs’ exposure to COVID-19 patients. Lastly, a standardized questionnaire will allow epidemiological comparison across various research sites.

## 5. Conclusions

Our study, part of the WHO project “*Assessment of risk factors for coronavirus disease 2019 (COVID-19) in health workers: protocol for a case-control study*” evaluated the personal, social and occupational factors associated with SARS-CoV-2 infection among a cohort of 120 HCWs exposed to COVID-19 patients in the two teaching hospitals of Trieste (North-eastern Italy). Non-significant risk factors for SARS-CoV-2 infection were found, but very high knowledge and adherence to IPC measures featured the population of the present study. The lack of significant results may be due to low statistical power or infections attributable to sources not evaluated by the present survey instrument, e.g., contact between positive asymptomatic HCWs during breaks or imperfect PPE donning. In particular, longer COVID-9 training was associated with lower risk of SARS-CoV-2 infection at univariable analysis. A larger study sample might be able to confirm the latter result also at multivariable analysis.

Further research is recommended to better elucidate and evaluate risk factors for SARS-CoV-2 infection in HCWs. The multi-centric WHO study relying on a bigger sample should be enough powerful to shed light on this still poorly understood issue, supporting the design of public health policies aimed at containing the spread of COVID-19 in health care settings.

## Figures and Tables

**Table 1:** Distribution of the study population by cases, controls and explanatory factors. Number (N) and column percentages (%) and corresponding *p* value (Mann–Whitney for linear terms and chi-square for categorical terms).

Factors		Cases N = 29 (24.2)	Controls N = 91 (75.8)	Total N = 120	*p*-Value
**Sex**	**Female**	14 (48.3)	56 (61.5)	70 (58.3)	0.211
	**Males**	15 (51.7)	35 (38.5)	50 (41.7)	
**Age (median; IQR)**		42 (30; 53)	40 (33; 52)	40 (32; 52)	0.990
**Occupation**	**Medical doctor**	5 (17.2)	8 (8.8)	13 (10.8)	0.673
	**Registered nurse**	14 (48.3)	46 (50.5)	60 (50.0)	
	**Assistant nurse**	4(13.8)	21 (23.1)	25 (20.8)	
	**Radiology technician**	1 (3.4)	3 (3.3)	4 (4.4)	
	**Physiotherapist**	0 (0)	2 (2.2)	2 (1.7)	
	**Admission clerk**	0 (0)	1 (1.1)	1 (0.8)	
	**Patient transporter**	5 (17.2)	10 (11)	15 (12.5)	
**Educational Level**	**Secondary/Tertiary**	9 (31.0)	26 (28.6)	35 (29.2)	0.799
	**Graduate**	20 (69.0)	65 (71.4)	85 (70.8)	
**Ward type**	**Non-COVID-19**	16 (55.2)	55 (60.4)	71 (59.2)	0.518
	**COVID-19**	13 (44.8)	36 (39.6)	49 (40.8)	
**Number of days in a COVID-19 ward in previous 14 days—median (IQR)**		10 (7; 12)	10 (10; 12)	10 (10; 12)	0.783
**COVID-19 training**	**Not-received**	8 (27.6)	26 (28.6)	34 (28.3)	0.229
	**Received**	21 (72.4)	65 (71.4)	86 (71.7)	
**Duration of** **COVID-19 training** **(h)**	**≥2**	10 (34.5)	51 (56.0)	61 (50.8)	0.141
	**<2**	17 (58.6)	36 (39.6)	53 (44.2)	
	**Unknown**	2 (6.9)	4 (4.4)	6 (5–0)	
**Training method**	**Theoretical**	4 (13.8)	23 (25.3)	27 (22.5)	0.754
	**Practical**	12 (41.4)	35 (38.5)	47 (39.2)	
	**Theoretical & Practical**	10 (34.5)	27 (29.7)	37 (30.8)	
	**Non reported**	3 (10.3)	6 (6.6)	9 (7.5)	
**Public transport use** **in last 14 days**	**≥8 days**	4 (13.8)	14 (15.4)	18 (15.1)	0.337
	**4–7 days**	0 (0)	4 (4.4)	4 (3.4)	
	**<3 days**	1 (3.4)	11 (12.1)	12 (10.1)	
	**None**	24 (82.8)	62 (68.1)	86 (71.4)	
**Social interactions** **in last 14 days** **(Number)**	**≥8 days**	1 (3.4)	5 (5.5)	6 (5.0)	0.346
	**4–7 days**	1 (3.4)	8 (8.8)	9 (7.5)	
	**<3 days**	5 (17.2)	27 (29.7)	32 (26.7)	
	**None**	22 (75.9)	51 (56.0)	73 (60.8)	

**Table 2:** Knowledge of and adherence to infection prevention and control (IPC) measures among health care workers (HCWs). Number (N), column percentage (%) and corresponding chi-square *p*-value.

Questions		Cases	Controls	Total	*p*-Value
**Do you know the recommended moments of hand hygiene?**	**I don’t know them**	-	1 (1.1)	1 (0.8)	0.843
	**Yes, all 5**	28 (96.5)	87 (95.6)	115 (95.8)	
	**Yes, all 6**	1 (3.5)	3 (3.3)	4 (3.3)	
**Do you follow the recommended hand hygiene practices?**	**Always, as recommended**	29 (100)	91 (100)	120 (100)	NA
**Do you use alcohol-based hand rub or soap and water before touching a patient?**	**Always, as recommended**	29 (100)	90 (98.9)	119 (99.2)	0.571
	**Occasionally**	0 (0)	1 (1.10)	0 (0)	
**Do you use alcohol-based hand rub or soap and water before cleaning/aseptic procedures?**	**Always, as recommended**	29 (100)	90 (98.9)	119 (99.2)	0.571
	**Never**	-	1 (1.10)	1 (0.8)	
**Do you use alcohol-based hand rub or soap and water after (risk of) body fluid exposure?**	**Always, as recommended**	29 (100)	90 (98.9)	119 (99.2)	0.571
	**Never**	0 (0)	1 (1.10)	1 (0.8)	
**Do you use alcohol-based hand rub or soap and water after touching a patient?**	**Always, as recommended**	29 (100)	90 (98.9)	119 (99.2)	0.571
	**Never**	-	1 (1.10)	1 (0.8)	
**Do you use alcohol-based hand rub or soap and water after touching a patient’s surroundings?**	**Always, as recommended**	29 (100)	89 (97.8)	118 (98.3)	0.723
	**Most of the time**	0 (0)	1 (1.1)	1 (0.8)	
	**Never**	-	1 (1.1)	1 (0.8)	
**Is alcohol-based hand rub available at point of care?**	**Yes**	29 (100)	89 (97.8)	118 (98.3)	0.421
	**Occasionally**	-	2 (2.2)	2 (1.7)	
**Do you follow IPC standard precautions when in contact with any patient?**	**Always, as recommended**	29 (100)	91 (100)	120 (100)	NA
**Do you wear PPE when recommended?**	**Always, according to** **the risk assessment**	29 (100)	91 (100)	120 (100)	NA
**Is PPE available in sufficient quantity in the health care facility?**	**Yes**	28 (96.55)	88 (96.7)	116 (96.7)	0.968
	**No**	1 (3.45)	-	1 (0.8)	
	**Unknown**	-	3 (3.3)	3 (2.5)	

**Table 3:** Characteristics of HCWs’ exposure to COVID-19 patients between cases and control. Number (N), median, Interquartile range (IQR), column percentage (%) and Mann–Whitney (for continuous terms) or chi-square (for categorical terms) *p*-value.

Factors			Cases (N = 29)	Controls (N = 91)	Total (N = 120)	*p*-Value
**Close contacts < 1 m**		**No**	5 (17.2)	11 (12.1)	16 (13.3)	0.376
		**Yes**	24 (82.8)	80 (87.9)	104 (86.7)	
**% of close contacts**	**Number of close contacts in last 14 days**	**<10**	7 (29.2)	23 (28.7)	30 (28.9)	0.851
		**10–50**	2 (8.3)	10 (12.5)	12 (11.5)	
		**>50**	15 (62.5)	47 (58.8)	62 (59.6)	
	**Maximum amount of time spent in close contact with COVID-19 patients in last 14 days**	**<5 min**	1 (4.2)	8 (10.0)	9 (8.6)	0.788
		**5–15 min**	2 (8.3)	5 (6.2)	7 (6.7)	
		**>15 min**	21 (87.5)	67 (83.8)	88 (84.6)	
	**Face-to-face exposure in last 14 days**	**No**	4 (16.7)	20 (25.0)	24 (23.1)	0.430
		**Yes**	20 (83.3)	60 (75)	80 (76.9)	
**% of face-to-face exposure**	**DPI usage during face-to-face exposure**	**No**	0	0	0	NA
		**Yes**	20 (100)	60 (100)	80 (100)	
	**Test-fitted respirator**	**No**	3 (15.0)	1(5.0)	12 (15.0)	0.893
		**Yes**	17 (85.0)	51 (85.0)	68 (85.0)	
	**Gloves removal**	**No**	1 (5.0%)	4 (6.7)	5 (6.2)	0.839
		**Yes**	19 (95.0)	56 (93.3)	75 (93.8)	
**Exposure to aerosol-generating procedures**		**No**	21 (72.4)	44 (48.4)	65 (54.2)	0.021
		**Yes**	8 (27.6)	47 (51.6)	55 (45.8)	
**Exposure to COVID-19 patients’ body fluids**		**No**	17 (58.6)	39 (39.5)	56 (46.7)	0.267
		**Yes**	12 (41.3)	52 (60.5)	64 (53.3)	
**Exposure to COVID-19 patients’ Materials**		**No**	6 (20.7)	17 (18.7)	23 (19.2)	0.086
		**Yes**	23 (79.3)	74 (81.3)	97 (80.8)	
**Exposure to COVID-19 patients’ surroundings**		**No**	2 (6.9)	12 (13.2)	14 (11.7)	0.076
		**Yes**	27 (93.1)	79 (86.8)	106 (88.3)	

**Table 4:** Clinical profile of COVID-19-infected HCWs. Number (N) and column percentage (%).

SYMPTOMS	MALES	FEMALES	Total
**Fever**	6 (40)	8 (57.1)	14 (48.3)
**Sore throat**	3 (20)	3 (21.4)	6 (20.7)
**Cough**	8 (53.3)	6 (42.9)	14 (48.3)
**Runny nose**	4 (26.7)	3 (21.4)	7 (24.1)
**Shortness of breath**	1 (6.7)	0 (0)	1 (3.4)
**Chills**	2 (13.3)	2 (14.3)	4 (13.8)
**Vomiting**	1 (6.7)	1 (7.1)	2 (6.9)
**Nausea**	2 (13.3)	1 (7.1)	3 (10.3)
**Diarrhea**	1 (6.7)	1 (7.1)	2 (6.9)
**Headache**	1 (6.7)	5 (35.7)	6 (20.7)
**Rash**	0 (0)	0 (0)	0 (0)
**Conjunctivitis**	0 (0)	0 (0)	0 (0)
**Myalgia**	8 (53.3)	5 (35.7)	13 (44.8)
**Joint ache**	1 (6.7)	1 (7.1)	2 (6.9)
**Loss of appetite**	0 (0)	1 (7.1)	1 (3.4)
**Anosmia/dysgeusia**	7 (46.7)	5 (35.7)	12 (41.4)
**Nosebleed**	0 (0)	0 (0)	0 (0)
**Fatigue**	1 (6.7)	6 (42.9)	7 (24.1)
**No symptoms**	1 (6.7)	0 (0)	1 (3.4)

**Table 5:** Medical history and medication intake of health care workers. Number (N), column percentage (%) and *p* value.

Factors			Cases	Controls	Total	*p*-Value
**Any underlying disease or pre-existing condition(s)**	**TOTAL**	**No**	19 (65.5)	69 (75.80	88 (73.3)	0.229
		**Yes**	10 (34.5)	22 (24.2)	32 (26.9)	
	**Pregnancy**		0 (0)	1 (1.1)	1 (0.8)	
	**Obesity**		1 (3.5)	1 (1.1)	2 (1.7)	
	**Cancer**		2 (6.9)	0 (0)	2 (1.7)	
	**Diabetes**		0 (0)	0 (0)	0 (0)	
	**HIV/immunodeficiency**		0 (0)	0 (0)	0 (0)	
	**Cardiac disease**		0 (0)	2 (2.2)	2 (1.7)	
	**Asthma**		1 (3.5)	6 (6.6)	7 (5.8)	
	**Chronic lung disease**		0 (0)	0 (0)	0 (0)	
	**Chronic liver disease**		0 (0)	0 (0)	0 (0)	
	**Chronic hematological disorder**		0 (0)	0 (0)	0 (0)	
	**Chronic kidney disease**		1 (3.5)	1 (1.1)	1 (0.8)	
	**Chronic neurological disease**		0 (0)	0 (0)	0 (0)	
	**Organ/bone marrow recipient**		0 (0)	0 (0)	0 (0)	
	**Other**		8 (27.6)	20 (22)	28 (23.3)	
**Any regular** **medication intake**	**TOTAL**	**No**	19 (65.5)	65 (71.4)	84 (70.0)	0.472
		**Yes**	10 (34.5)	26 (28.6)	36 (30.0)	
	**Statin**		0 (0)	3 (3.3)	3 (2.5)	
	**Steroid**		0 (0)	1 (1.1)	1 (0.8)	
	**Antidiabetic**		0 (0)	0 (0)	0 (0)	
	**Immunosuppressive**		1 (3.5)	1 (1.1)	2 (1.7)	
	**Other**		10 (35.7)	25 (27.5)	35 (29.2)	

**Table 6:** Distribution of chemoluminescence Immunoassay (CLIA) serology (cut-off ≥ 33.8 BAU/mL) and enzyme-linked immunosorbent assay (ELISA) (AU/mL ≥ 1) among cases and controls. Median, interquartile range (IQR) and *p*-value of the Mann–Whitney test. CLIA at T1 performed on 27/29 cases (2 of them testing positive) and on 91/91 controls (3 of them testing positive). CLIA at T2 performed on 29/29 cases (all testing positive) and on 84/91 controls (4 of them testing positive). Missing = missing information.

TEST	CASES	CONTROLS	TOTAL SAMPLE	*p*
**CLIA T1** (Median; IQR)	22.4 (21.4; 23.4) (Missing: 2)	23.6 (13.7; 29.4) (Missing: 0)	23.4 (21.4; 23.6) (Missing: 2)	0.020
**CLIA T2** (Median; IQR)	50.6 (27.2; 89.9) (Missing: 0)	23.0 (17.9; 34.4) (Missing: 7)	47.0 (26.8; 67.2) (Missing: 7)	
**ELISA T1** (Median; IQR)	0.010 (0.006; 0.013) (Missing: 23)	0.011 (0.006; 0.046) (Missing: 1)	0.010 (0.006; 0.044) (Missing: 24)	<0.001
**ELISA T2** (Median; IQR)	2.589 (1.433; 3.168) (Missing: 6)	0.046 (0.024; 0.153) (Missing: 32)	0.111 (0.027; 1.360) (Missing: 38)	

**Table 7:** Univariable and multivariable logistic regression analysis investigating the risk of SARS-CoV-2 infection among health care workers of the two teaching hospitals of Trieste. Odds ratios unadjusted (OR) and adjusted (aOR) with 95% confidence interval (95% CI). Significant estimates are marked in bold. Multivariable logistic model fitted on 82 complete case (analysis) observations.

Factors		Univariable OR (95% CI)	Multivariable OR (95% CI)
**Sex**	**Males**	*Reference*	*Reference*
	**Females**	0.58 (0.25; 1.35)	0.66 (0.21; 2.03)
**Age (years) (linear term)**		1.00 (0.96; 1.04)	0.98 (0.93; 1.03)
**Occupation**	**Medical doctor**	*Reference*	
	**Registered nurse**	0.49 (0.14; 7.29)	
	**Assistant nurse**	0.30 (0.06; 1.43)	
	**X-ray technician**	0.53 (0.04; 6.60)	
	**Patient transporter**	0.80 (0.17; 3.76)	
**Educational level**	**Secondary/** **Tertiary**	*Reference*	
	**Graduate**	0.89 (0.35; 2.20)	
**Ward Type**	**Non-COVID-19**	*Reference*	
	**COVID-19**	1.32 (0.56; 3.10)	
**COVID-19 wards dedicated days**		0.96 (0.82; 1.12)	
**Duration of COVID-19 training (hours)**	**<2**	*Reference*	*Reference*
	**≥2**	**0.41 (0.17–0.89)**	0.36 (0.11; 1.12)
**Public transport use, last 14 days**	**No**	*Reference*	
	**Yes**	0.56 (0.20; 1.52)	
**Social interactions, in last 14 days**	**No**	*Reference*	
	**Yes**	0.49 (0.19; 1.21)	
**Close contact < 1 m**	**No**	*Reference*	
	**Yes**	1.1 (0.33; 3.31)	
**Exposure to aerosol-generating procedures**	**No**	*Reference*	
	**Yes**	0.50 (0.21; 1.19)	
**Exposure to COVID-19 patients’ body fluids**	**No**	*Reference*	
	**Yes**	0.60 (0.24; 1.48)	
**Exposure to COVID-19 patients’ materials**	**No**	*Reference*	
	**Yes**	2.32 (0.80; 6.69)	
**Exposure to COVID-19 patients’ surroundings**	**No**	*Reference*	
	**Yes**	6.29 (0.75; 52.0)	
**ELISA values** (optical density) (linear)		**8.7 (3.49; 21.77)**	**3.8 (1.71–8.44)**

## Data Availability

The datasets generated and analyzed during the current study are not publicly available, since they were purposively collected by the authors for the present study, but may be available from the corresponding author on reasonable request.
